# Hominoid chromosomal rearrangements on 17q map to complex regions of segmental duplication

**DOI:** 10.1186/gb-2008-9-2-r28

**Published:** 2008-02-07

**Authors:** Maria Francesca Cardone, Zhaoshi Jiang, Pietro D'Addabbo, Nicoletta Archidiacono, Mariano Rocchi, Evan E Eichler, Mario Ventura

**Affiliations:** 1 Department of Genetics and Microbiology, University of Bari, Via Amendola, Bari, 70126, Italy; 2 Department of Genome Sciences, University of Washington School of Medicine and the Howard Hughes Medical Institute, NE Pacific Street, Seattle, Washington, 98195, USA; 3 Howard Hughes Medical Institute, NE Pacific Street, Seattle, Washington, 98195, USA

## Abstract

**Background:**

Chromosomal rearrangements, such as translocations and inversions, are recurrent phenomena during evolution, and both of them are involved in reproductive isolation and speciation. To better understand the molecular basis of chromosome rearrangements and their part in karyotype evolution, we have investigated the history of human chromosome 17 by comparative fluorescence *in situ *hybridization (FISH) and sequence analysis.

**Results:**

Human bacterial artificial chromosome/p1 artificial chromosome probes spanning the length of chromosome 17 were used in FISH experiments on great apes, Old World monkeys and New World monkeys to study the evolutionary history of this chromosome. We observed that the macaque marker order represents the ancestral organization. Human, chimpanzee and gorilla homologous chromosomes differ by a paracentric inversion that occurred specifically in the *Homo sapiens/Pan troglodytes/Gorilla gorilla *ancestor. Detailed analyses of the paracentric inversion revealed that the breakpoints mapped to two regions syntenic to human 17q12/21 and 17q23, both rich in segmental duplications.

**Conclusion:**

Sequence analyses of the human and macaque organization suggest that the duplication events occurred in the catarrhine ancestor with the duplication blocks continuing to duplicate or undergo gene conversion during evolution of the hominoid lineage. We propose that the presence of these duplicons has mediated the inversion in the *H. sapiens/P. troglodytes/G. gorilla *ancestor. Recently, the same duplication blocks have been shown to be polymorphic in the human population and to be involved in triggering microdeletion and duplication in human. These results further support a model where genomic architecture has a direct role in both rearrangement involved in karyotype evolution and genomic instability in human.

## Abstract 2

A FISH analysis of chromosome 17 homologs in primates suggests that genomic architecture has a direct role in karyotype evolution and in the genomic instability associated with human disease.

## Background

Karyotype evolution was first studied by classical cytogenetics based on comparison of banding pattern and, more recently, using molecular cytogenetic tools such as fluorescence *in situ *hybridization (FISH). Chromosome painting and reciprocal chromosome painting, in particular, have delineated the organization of the karyotype in the primate ancestor [1,2] and in the mammalian ancestor [3-7]. Chromosome painting libraries alone cannot detect marker order arrangement along chromosomes. In contrast, bacterial artificial chromosome (BAC) clones in FISH experiments, defining the marker order, can be used in phylogenetically related species to reconstruct the evolutionary history of chromosomal rearrangements in extant primates [8,9]. FISH experiments are dependent upon the genetic distance between species. Species-specific BAC clones can be helpful in this regard. If sequence data are available, overgo probes (two 24-mer oligonucleotides that share eight base pairs of complementary sequence at their 3' ends) or specific sequence tagged sites (STSs) are designed in conserved regions of a reference species to recover orthologous clones by library hybridization [10]. Such orthologous probes facilitate comparison between more distant species and the definition of breakpoints. Molecular analysis of breakpoint regions can provide insight into mechanisms involved in karyotype evolution [11,12].

Recently, a nearly complete sequence of human chromosome 17 was obtained as part of the Human Genome Project. The chromosome sequence is interrupted by nine euchromatic gaps and one gap corresponding to the centromere [13]. It is one of the richest chromosomes in terms of G+C and gene content. It is also significantly enriched in segmental duplications, ranking fourth in duplication density after chromosomes Y, 22 and 16 [13-19].

Many of the blocks of intrachromosomal segmental duplication on 17p have already been described as involved in DNA rearrangements associated with several well-studied microdeletion disorders. These include hereditary neuropathy with pressure palsies at 17p12, Smith-Magenis syndrome deletions at 17p11.2 [20-22], and the microduplication at 17p12 in the case of Charcot-Marie-Tooth disease type 1A [23-26]. More recently, segmental duplication blocks on the long arm of chromosome 17 have been implicated in recurrent microdeletions associated with mental retardation [27-29], diabetes and renal disease [30]; further data have demonstrated the structural complexity of the region surrounding one multiple sclerosis susceptibility locus on chromosome 17q22-24 [20,31]. The complex architecture in this region is also responsible for susceptibility to one of the most common somatic rearrangement events characterized, isodicentric 17q, which is associated with several cancers of poor prognosis [32].

In the present paper, we describe the evolutionary history of human chromosome 17 in primates and a detailed study of the evolution of segmental duplications in 17q12 and 17q23. A total of 58 human BAC clones and 27 macaque specific BAC clones were used in FISH experiments on great apes and on representatives of Old World monkeys and New World monkeys in order to delineate the chromosome 17 phylogeny in primates using the domestic cat and mouse genome sequences as representative non-primate mammalian outgroups. We characterized the paracentric inversion breakpoints by FISH and detailed sequence analyses, which show a clear association between inverson breakpoints and intrachromosomal segmental duplication blocks. The assignment of the breakpoint region to clusters of segmental duplications furthers the claim that genomic architecture is a significant factor in hominoid karyotype evolution [33-36].

## Results

### Evolutionary history of human chromosome 17

Chromosome 17 evolution was studied, initially, by two-color FISH of 12 single copy human BAC clones evenly distributed along the chromosome (Table 1). The probes were hybridized on metaphase chromosomal spreads of great ape species (chimpanzee (*Pan troglodytes*), gorilla (*Gorilla gorilla*) and orangutan (*Pongo pygmaeus*)), rhesus macaque (*Macaca mulatta*) as a representative Old World monkey, and three New World monkeys (marmoset (*Callithrix jacchus*), dusky titi (*Callicebus moloch*) and woolly monkey (*Lagothrix lagothricha*)). We performed a parallel analysis on *Felix catus *to serve as a mammalian outgroup. We designed 'overgo' probes corresponding to conserved sequence within each human BAC probe [10] and retrieved corresponding large insert genomic clones by hybridization against a cat genomic BAC library (RP-86). This approach facilitated comparative mapping by assembling a panel of cat probes orthologous to each of the human BAC loci (Additional data file 1). The cat clones were used in FISH experiments on metaphase spreads of *F. catus *and marker order was determined. In addition, we also compared the organization of human chromosome 17 and the finished sequence of the mouse (*Mus musculus*) orthologue (chromosome 11), the first finished mouse chromosome [13], in an effort to identify the likely mammalian ancestral state. If the centromere position is excluded, then orangutan, rhesus macaque and the New World monkeys share the same marker order. *F. catus *differs from this form for an inversion between the markers A and B. Zody *et al*. [13] have reported the same marker order arrangement on mouse chromosome 11.

**Table 1:** Relevant BAC clones used in the study

Code	Name	Accession number	Chromosome band	Mapping (UCSC March 2006)
A	RP11-411G7	AC027455	17p13.3	chr17:427,025-572,435
A1*	*RP11-769H22*	*BES*	*17p13.3*	*chr17:7*,*849*,*179-8*,*011*,*176*
A2**	*RP11-385D13*	*AC005838*	*17p13.3*	*chr17:15*,*367*,*740-15*,*435*,*530*
B	RP11-367G9	AC079111	17p11.2	chr17:16,853,117-17,016,545
Cen				**chr17:22,187,134-22,287,133**
C	RP11-28A22	AC005691	17q11.2	chr17:29,842,523-29,999,343
D	RP11-212E8	AC005552	17q11.2	chr17:30,009,726-30,175,558
E	RP11-115K3	AC113211	17q12	chr17:33,140,726-33,322,352
*E1*	*RP11-932C2*	*AC124789*	*17q12*	*chr17:33*,*713*,*298-33*,*818*,*972*
F	RP11-15D16	BES	17q21.31	chr17:42,268,624-42,438,970
G	RP11-456D7	AC027152	17q21.31	chr17:43,587,728-43,720,471
G1*	*RP5-1029K10*	*AC006487*	*17q21.32*	*chr17:44*,*918*,*039-45*,*104*,*642*
H	RP11-170D6	AC091154	17q22	chr17:48,430,427-48,606,237
I	RP11-758H9	AC091271	17q23.2	chr17:55,012,846-55,148,629
J	RP11-42F20	AC008158	17q23.2	chr17:57,449,286-57,597,398
*J1*	*RP11-465I18*	*BES*	*17q23.2*	*chr17:57*,*474*,*072-57*,*660*,*715*
*J2*	*RP11-50G1*	*BES*		*chr17:57*,*765*,*687-57*,*954*,*835*
K	RP11-450M16	AC073299	17q23.3	chr17:59,588,364-59,747,225
L	RP13-650J16	AC105341	17q25.3	chr17:77,541,734-77,680,864
End				**chr17:78,599,126-78,738,256**

**Figure 1:**
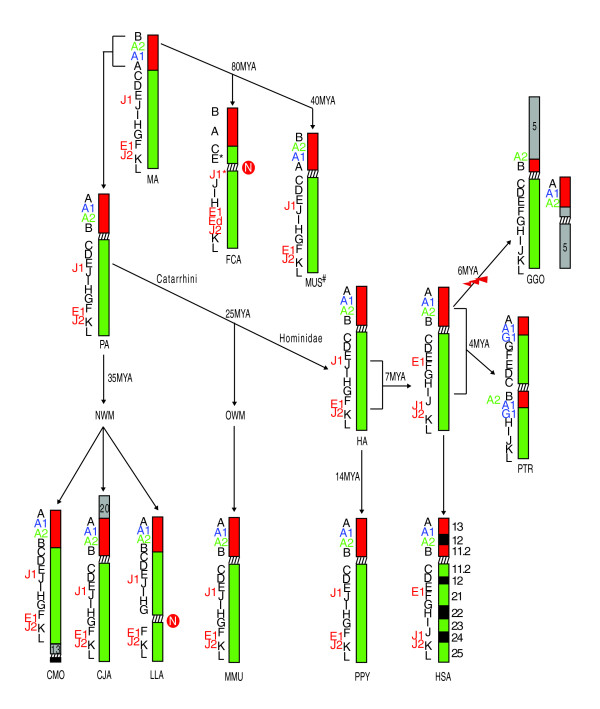
Diagrammatic representation of the evolutionary history of chromosome 17. Marker order arrangement in the studied species, from which the arrangements of the mammalian ancestor (MA) and primate ancestor (PA) were derived (see text). The black letters on the left of each chromosome refer to the panel of BAC probes used in FISH experiments and reported in Table 1. Letters on the *F. catus *(FCA) chromosome refer to BACs reported in Additional data file 2 obtained by library screening. E* and J1* indicate the cat probes obtained by library screenings and corresponding to human E and J1 probes. The hash symbol for *M. musculus *(MUS) indicates the arrangement was derived from Zody *et al*. [13]. Letters in green or blue indicate BAC probes derived from literature data (see text for details). In red are additional BACs used to delimit the breakpoints or those that yielded duplicated signals. The time of divergence is reported near the arrow. The 'N' in the red circle indicates an evolutionary neocentromere. CJA, *Callitrix jacchus*; CMO, *Callicebus moloch*; GGO, *Gorilla gorilla*; HA, hominoid ancestor; HSA, *Homo sapiens*; LLA, *Lagotrix lagotricha*; MMU, *Macaca mulatta*; NWM, New World monkey; OWM, Old World monkey; PPY, *Pongo pygmeus*; PTR, *Pan troglodytes*. Red and green regions indicate human short and long arm respectively; black bands are the Giemsa cytobands of chromosome 17, letters in color reported further BAC probes used to refine breakpoints (see the text for details) and gray segments and numbers report the human chromosomes sharing sintenic association with chromosome 17.

Probes in regular type (12) were used to characterize all primate species. Probes in italics were used to define specific rearrangements. Asterisks indicate probes used to confirm literature data (*P. pygmeus breakpoints, **G. gorilla breakpoints; for details, see the text and Figure 1). Cen, Centromere; End, long arm telomere. Bold indicates the relative cytogenetic positions in the chromosome 17 to focus their localization among the other markers.

The macaque organization, therefore, could be considered to represent the ancestral hominoid organization, with a paracentric inversion of the long arm responsible for the current human chromosome 17. During our FISH analysis, we narrowed the breakpoints of this rearrangement between FISH marker probes E and F and between marker probes J and K (Table 1 and Additional data file 2). Our analysis confirmed the location of the pericentric inversion in chimpanzee and chromosomal translocation in gorilla [11,37]. Moreover, we found evidence of two centromere repositioning events [38,39] in *F. catus *and *L. lagotricha*. Figure 1 schematically summarizes our FISH results and the most parsimonious chromosomal changes necessary to reconstruct chromosome 17 evolution in primates.

### Breakpoint definition and analysis

**Figure 2:**
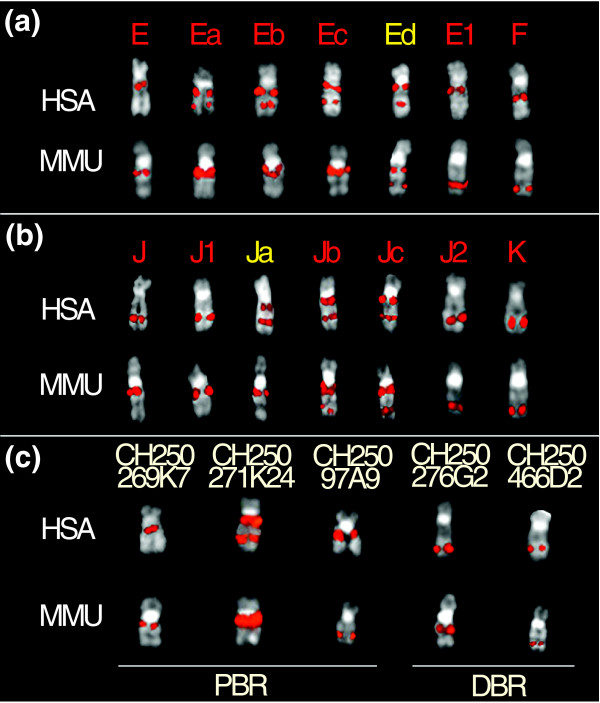
Examples of hybridization experiments on macaque (MMU) and human (HSA) with BAC clones in the breakpoint regions. **(a) **FISH results for the PBR: under the chromosomes are reported the letter codes assigned to each BAC as reported in Additional data file 1 and Figure 1. BACs that yielded single signal both on MMU and HSA are listed in white, duplicated clones in MMU and HSA are listed in red, and duplicated BACs but with a different pattern of hybridization are listed in yellow. **(b) **Results for the distal breakpoint region (DBR). **(c) **Examples of hybridization on MMU and HSA with specific macaque BAC clones obtained by library screening or by *in silico *analyses (for detail see the text) for both the PBR and DBR.

The most basic approach to investigate the molecular mechanism underlying evolutionary chromosomal rearrangements is to characterize the breakpoint regions at the molecular level. We used two different approaches to define and analyze the breakpoint regions of the paracentric inversion in 17q. First, we further refined the proximal and distal breakpoints using additional human BAC probes, between marker probes E and F and between marker probes J and K. We tested 23 BACs against human and macaque in search of a clone that produced a single signal on human chromosome 17 and a double signal on both sides of the inverted segment in macaque (termed a split signal). As a result, we were able to localize the proximal breakpoint region (PBR) between the BACs RP11-115K3 (E, chr17:33,140,726-33,322,352) and RP11-923C2 (E1, chr17:33,713,298-33,818,972). Both the BACs gave a single signal in 17q12 on human metaphases. In contrast, in the macaque, E gave a single signal in a proximal not inverted position while E1 gave a signal in a distal position and it was thus included in the inverted segment (Figure 2a).

We refined the distal breakpoint region (DBR) between RP11-465I18 (J1, chr17:57,474,072-57,660,715) and RP11-50G1 (J2, chr17:57,765,687-57,954,835). Probe J1 gave a signal on 17q23 in human, but it was mapped proximally in macaque, while the BAC J2 produced a FISH signal in a distal location in both human and macaque, thus suggesting it was external to the inverted segment (Figure 2b). The most informative probes are reported in Additional data file 2 and the overall results are reported in Figure 1.

Complex duplication blocks mapping to the interval in E-E1 (390 kb chr17:33,322,352-33,713,298, named DUPA) and J1-J2 (100 kb chr17:57,660,715-57,765,687, named DUPB) complicated precise definition of the inversion breakpoints using this strategy. Four human BAC clones (Ea, Eb, Ec and Ed) spanning DUPA were tested by FISH on human and macaque. Each BAC gave duplicated signals on human chromosome 17 on both sides of the inverted segment. Notably, they also showed duplicated signals in *M. mulatta *in the proximal region except for the clone RP11-678G7 (Ed), which gave double signals in proximal and distal positions also on the macaque homologous chromosome (Figure 2a and Additional data file 2). Likewise, we tested three overlapping BACs (Ja, Jb, and Jc) spanning the distal DUPB breakpoint region by FISH. The BAC clone RP11-473H20 (Ja) showed duplicated signals in human 17q12 and 17q23, with duplicated signals present only in a proximal position in macaque. The remaining two clones gave double signals on both sides of the inverted segment in human and macaque (Figure 2b and Additional data file 2).

To further refine inversion breakpoints and duplication organization, we used a panel of 27 macaque-specific clones obtained from the macaque library by *in silico *[40] and overgo-probes/STS library screenings. Overlapping clones covering the PBR and DBR were assembled by end-sequence similarity searches against the human genome (Additional data file 3). All clones were tested by FISH.

One overgo probe, BP1/BP2 (chr17:33,612,761-33,612,796) was designed from human sequence for the PBR. We obtained only two positive clones mapping in the inverted segment (green in Additional data file 3). Gaps in the assembled human sequence prevent the further design of more useful overgo probes. BAC clone CH250-269k7 (AC140608) was mapped by BLAST sequence similarity searches, proximally (located closer to the centromere) respect to the overgo probe BP1/BP2. It produced the same hybridization pattern of the human E clone (not inverted clone) on human 17q12 and on the orthologous region in macaque. It is noteworthy that other macaque BAC clones covering the PBR gave duplicated signals both in proximal and distal regions on human chromosome 17 and proximally in macaque, thus preventing the further refinement of the PBR by FISH (Figure 2c and Additional data file 3).

We designed two STSs to the DBR: 20g10-51l5 (chr17:57,729,109-57,729,131) and SHGC-78807 (chr17:57,826,604-57,826,825). Three positive clones were obtained using 20g10-51l5 STS (blue in Additional data file 3) and further tested by FISH. They produced signals on human 17q23 and proximally on macaque, internal to the inversion. In addition, three BAC clones obtained by SHGC-78807 screening produced FISH signals distally both in human and macaque, thus mapping external to the inversion (Additional data file 3). BAC clone CH250-466D2 (only one end mapped by BLAST against human to chr17:57,734,101-57,734,926 distal to the 20g10-51l5 STS) produced signals distally in both human and macaque.

Combined, our approaches allowed us to further refine the PBR to an approximately 290 kb region between human BAC clone E, RP11-115K3 (33,322,352) and the overgo probes BP1/BP2 (33,612,761) and the DBR to an approximately 5 kb region between STS 20g10-51l5 (57,729,131) and the macaque BAC CH250-466D2 (57,734,101).

### Organization and evolution of segmental duplications

**Figure 3:**
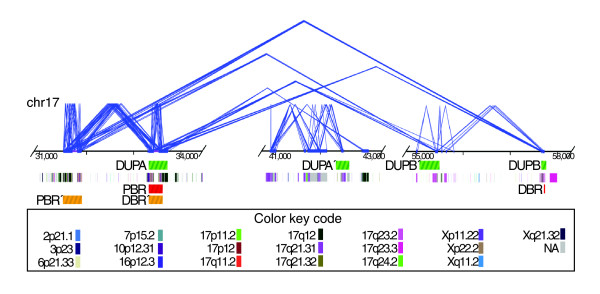
Duplication architecture in 17q21-23. Structure of duplication regions: large (≥10 kb), high identity (≥95%) segmental duplications are shown between DUPA, DUPA', DUPB and DUPB' (green-striped blocks in the first row) as pairwise alignments (blue lines). Underlying duplicon mosaic structure (second bottom row) was defined using the program DupMasker [56]. The different colors represent the different cytoband locations for the ancestral loci of duplications (see the color key code below; NA, ancestral locus not determined) [56]. DUPA-DUPB' share fewer high-identity duplications compared to DUPA-DUPB (total aligned = 104.7 kb, average identity = 97.4%; Additional data file 6). The PBR and DBR are shown as red-striped blocks (third row). The proximal and distal breakpoint regions found in microdeletion cases (PBR' and DRB', fourth row), according to Mefford *et al*. [30], are shown as orange-striped blocks.

We performed a series of bioinformatics and comparative FISH analyses to provide further insight into the evolutionary history of the duplication blocks mapping to the PBR and DBR. Human duplication blocks are typically organized as mosaic structures composed of duplications of diverse evolutionary origin [41]. We first considered the evolutionary architecture of each of the regions based on a recently developed algorithm designed to delineate the most likely ancestral duplication events (duplicons) within each block (Figure 3). *In silico *analysis was performed in the regions flanking the PBR and DBR and two more duplication clusters: DUPA' (chr17:42,332,487-42,612,217) and DUPB' (chr17:55,074,551-55,498,114) mapping between proximal and DBRs. Each duplicated block, DUPA, DUPA', DUPB' and DUPB showed a very complex organization, having unique and shared duplicon modules. The most common duplicon corresponded to the rapidly evolving TBC1D3 gene family, which mapped multiple times to DUPA, DUPB and DUPB'. We found four copies in DUPA, one in DUPB' and two entire copies of the gene in DUPB. Interestingly, by comparing the different copies of this gene both inside each block of duplication and between the two clusters, we found that the sequence similarity was higher inside each cluster (approximately 90%) than between DUPA and DUPB (87-88%). In addition, sequence analyses showed copies of the same gene, or part of it, also in 17p11/12 with a lower sequence similarity (85-86%). Similar analyses were performed on the Rhesus genome in the UCSC browser. We found five copies of the TBC1D3 gene mapping in the region orthologous to 17q12, where the PBR was previously defined, but no homologous region was detected in the orthologous region of 17q23.

**Figure 4:**
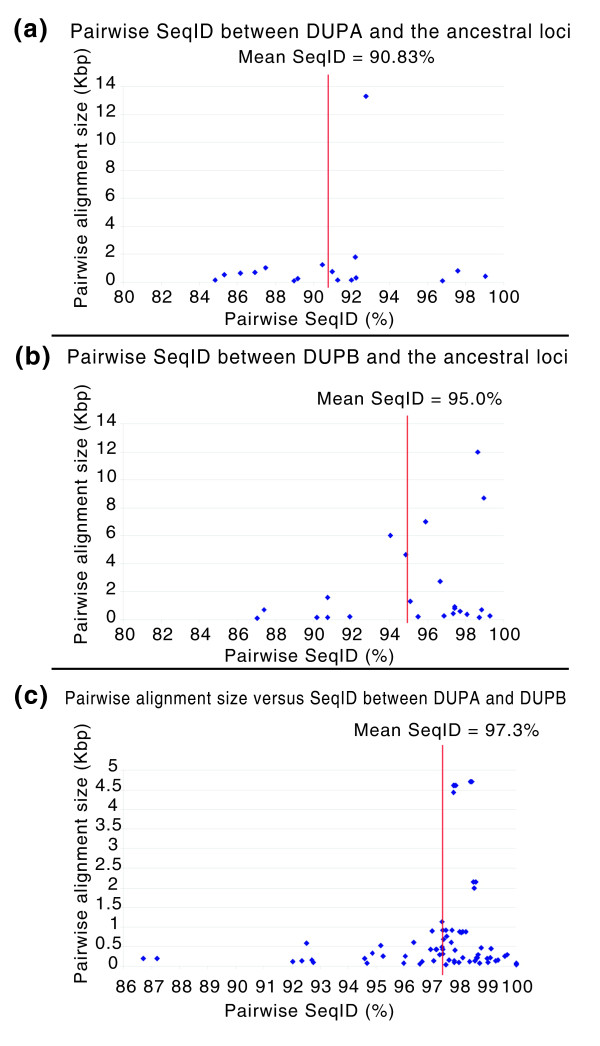
Pairwise alignment between DUPA and DUPB and their ancestral loci. **(a,b) **Pairwise sequence comparison between the ancestral loci and DUPA (a) and DUPB (b). We used ancestral state information provided by our previous study [56] and computed the pairwise alignment sequence identity (SeqID) between DUPA and DUPB and their putative ancestral loci. The alignment size is shown as the function of pairwise sequence identity between DUPA and DUPB versus their ancestral loci. **(c) **The alignment size is illustrated as the function of pairwise sequence identity between DUPA and DUPB. The average pairwise alignment between DUPA and DUPB is 97.3% (highlighted by red vertical line).

To estimate the age of each duplicon within DUPA and DUPB, we analyzed the sequence identity between derived duplicons mapping within each duplication block and the presumptive ancestral loci. Within DUPA, we found that most of the duplicons showed 85-99% (mean 90.83%) sequence identity to their ancestral loci (Figure 4a), while in DUPB a shorter range of sequence identity was observed (range 87-99%; mean 95.0%; Figure 4b). Assuming a relative neutral molecular clock of evolution and the average degree of sequence identity between macaque and human (94.5%) [42], these data suggest that duplicative transposition events began to form these regions prior to the separation of human and macaque lineages in a common catarrhine ancestor. Our data further suggest that DUPA may be more ancient, composed of duplicons that show greater divergence with respect to ancestral loci when compared to DUPB. Surprisingly, an analysis of pairwise alignments between DUPA and DUPB show many segments with a high degree of sequence identity (97-99%), perhaps as a result of gene conversion or recurrent reciprocal duplication events (Figure 4c). We note, however, that these high-identity alignments between DUPA and DUPB are significantly shorter when compared to ancestral-derivative duplication events. We found no evidence of segmental duplication between DUPA' and DUPA or between DUPB' and DUPB.

In order to further define the organization and the evolutionary history of duplication blocks, we performed a series of comparative FISH experiments on great ape, Old World monkey and New World monkey metaphase and interphase chromosomes using human BAC clones corresponding to the duplication blocks (Additional data file 2). Complex hybridization patterns were observed in chimpanzee and gorilla, while the orangutan showed a pattern similar to macaque. The exact order and localization of signals were defined by FISH on interphase and stretched chromosomes. The overall results are reported in Additional data file 4. Comparative FISH analysis using the BAC clones covering DUPA' showed duplicated signals on human chromosome 17q, but not on macaque. BAC clone RP11-178C3, spanning DUPB', gave duplicated signals also on macaque in the region orthologous to 17q12 (Additional data files 2, 4 and 5).

In the New World monkey, only the BAC RP11-678G7 (Ed) mapping in DUPA gave double signals in the proximal and distal region (orange in Additional data file 4). In order to understand if the cross-hybridization signal was due to an existing duplication or a splitting signal, BAC clones flanking clones to Ed were tested. Proximal human BAC RP11-493E8 (Ec) produced signals corresponding to the orthologous 17q12 region external to the inversion. The distal BAC, RP11-923C2 (E1), gave a signal in an orthologous region to 17q23, mapping internal to the inversion. FISH experiments using BAC clones covering DUPA', DUPB' and DUPB did not detect any duplication in New World monkeys. Finally, overgo probes mapping in the duplicated regions were used to screen a cat genome BAC library for evidence of segmental duplications. No evidence of duplications was found. Zody *et al*. [13] have suggested a lower rate of duplication in mouse chromosome 11 orthologous to human chromosome 17, while a burst of segmental duplications in the primate lineage on chromosome 17 has been reported by She *et al*. [19] and Bailey *et al*. [14,41].

## Discussion

In the present paper, we report the evolutionary history of human chromosome 17, as well as a detailed evolutionary study of the cluster duplications in 17q12 and q23. By using probes distributed along chromosome 17 we have determined the detailed marker order in seven primate species using the cat genome as a representative mammalian outgroup. In all examined species, including mouse [13], the chromosome homologous to human chromosome 17 was a single syntenic group or a contiguous part of a larger chromosome. The chromosome organization found in cat differed from this organization by a single inversion between markers A and B, also reported in mouse by Zody *et al*. [13]. We suggest that this form may be assumed as ancestral to mammals (MA in Figure 1). The inversion between markers A and B was not found in any of the analyzed primate species, suggesting that the macaque organization represents the ancestral primate configuration (PA in Figure 1). Moreover, the present study revealed that a paracentric inversion occurred in the ancestor of *H. sapiens*/*P. troglodytes*/*G. gorilla *after divergence of the orangutan (*P. pygmaeus*). Other species-specific rearrangements were found in the chimpanzee and gorilla lineage, as already described by Kehrer-Sawatzki *et al*. [11] and Stankiewicz *et al*. [37], respectively (Figure 1).

The paracentric inversion breakpoint regions were accurately defined and analyzed. Using molecular cytogenetics and bioinformatics approaches, we mapped the proximal breakpoint to a 290 kb region and the distal breakpoint to an approximately 5 kb region. Precise definition was prevented due to the presence of highly duplicated sequences in these regions. Our analysis showed that both the PBR and DBR localize inside large duplication blocks, named DUPA (approximately 390 kb) and DUPB (approximately 100 kb) in 17q12 and 17q23, respectively.

Sequence and FISH analysis detected four duplication blocks in the human inverted region, named DUPA, DUPA', DUPB' and DUPB, showing high sequence similarity, with the exception of DUPA'. We found DUPA' duplicated only in human and gorilla, thus suggesting this duplication occurred in the *H. sapiens*/*P. troglodytes*/*G. gorilla *ancestor, but was deleted in the *P. troglodytes *lineage.

Interestingly, our results demonstrated that the PBR, mapping in DUPA, is duplicated also in macaque, while no duplications were found in the distal region of the macaque homologous chromosome. These findings are also consistent with the presence of ancestral pairwise alignments of greater sequence divergence (mean = 90%; Figure 4a). No duplications were found in New World monkeys. This strongly suggests that DUPA may be considered the original cluster and the first event of duplication that occurred in the macaque ancestor about 25 million years ago. A subsequent duplication event, mediated by duplicative transposition, may have occurred in the great ape ancestor, thus creating paralogous duplication blocks as revealed by the presence of both DUPA and DUPB in all the analyzed hominoids. As reported by Stankiewicz *et al*. [37] in gorilla and Sawatzki *et al*. [11] in chimpanzee, paralogous sequences can trigger inversions by non-allelic homologous recombination. As well, we hypothesize that DUPA and DUPB, as paralogous duplication blocks in 17q12-17q23, triggered the paracentric inversion in the *H. sapiens*/*P. troglodytes*/*G. gorilla *ancestor after the divergence of orangutan (12 million years ago) by non-allelic homologous recombination. However, sequence comparison demonstrated that the similarity is higher between DUPA and DUPB compared to ancestral duplication loci, thus demonstrating that some duplication events or gene conversion events occurred more recently during hominoid evolution.

Moreover, our data show DUPA, DUPB' and DUPB presumably derived from an ancestral sequence that was subjected to multiple events of duplication during primate evolution. In this regard, this supports the non-random distribution of the segmental duplication within regions of a chromosome, thus defining precisely 17q12 and 17q23 as duplication hubs or acceptor regions [41].

Additional further events of gene conversion and duplication could explain the hybridization pattern of great ape 17p, suggesting that extensive duplications occurred after the rearrangements in *H. sapiens*, *P. troglodytes* and *G. gorilla*. Sequence comparison between the 17q clusters and 17p duplicons suggests that the copies on the p arm originated via more recent duplication events, as suggested by Bailey *et al*. [41].

The cluster of duplications in 17q22-24 has previously been described as being associated with a multiple sclerosis susceptibility locus [20,31]. More recently, segmental duplications in 17q12 have been described as involved in the genesis of microdeletion associated with pediatric renal disease and epilepsy and fetal renal dysplasia. Detailed analysis of seven affected individuals with microdeletion [30] show distal breakpoints clustering in a region corresponding to the DUPA described in the present paper (Figure 3). Further, these segmental duplications are polymorphic in copy number and structure among unaffected individuals, thus showing high variability in the human population [30].

Recent data reported segmental duplications as predisposing elements in genomic disorders associated with chromosomal rearrangements [43-45]. Several cases have been reported where segmental duplications triggered chromosomal rearrangements during evolution and in the human population [34,46]. Other examples show that our findings may be applicable to a broad range of taxa. The evolutionary chromosome translocation 4;19 breakpoints in *G. gorilla*, for instance, have been associated with the Charcot-Marie-Tooth microduplication syndrome [37]. Furthermore, in humans segmental duplications correspond to the location of a latent evolutionary centromere [38,39]. This 'centromere' can be reactivated to a functional centromere when a chromosomal rearrangement carries an acentric fragment and a small marker chromosome is recovered. It is unknown if segmental duplications can trigger this reactivation, but clearly they cluster around regions of neocentromere formation and breakpoint regions [38,39]. All of these data further support the link between duplications and chromosomal rearrangements involved in both genome evolution and genomic disorders.

## Conclusion

The present study has tracked the evolution of human chromosome 17, showing specific paracentric inversion in the *H. sapiens*/*P. troglodytes*/*G. gorilla *ancestor. The molecular characterizaton of the inversion breakpoints pointed out the role of segmental duplication in evolutionary rearrangements. Furthermore, the results defined important aspects of, and the relationship between, the role of segmental duplications in evolution and human genomic instability.

In summary, our molecular and computational analysis has revealed that genome architecture has evolved in a complex manner involving serial segmental duplications and gene conversion events that triggered evolutionary inversions. These regions are involved in human genomic instability, further supporting the role of segmental duplication in both evolution and human diseases.

## Materials and methods

### Cell lines

Metaphase preparations were obtained from cell lines (lymphoblasts or fibroblasts) from the following species. Great apes: common chimpanzee (*P. troglodytes*), gorilla (*G. gorilla*), and Borneo orangutan (*P. pygmaeus pygmaeus*). Old World monkeys: rhesus macaque (*M. mulatta*, Cercopithecinae). New World monkeys: wooly monkey (*L. lagothricha*, Atelinae), common marmoset (*C. jacchus*, Callitrichinae), and dusky titi (*C. moloch*, Callicebinae); and cat (*F. catus*).

### FISH experiments

DNA extraction from BACs was previously reported [38]. FISH experiments were performed essentially as described by Lichter *et al*. [47]. Digital images were obtained using a Leica DMRXA2 epifluorescence microscope equipped with a cooled CCD camera (Princeton Instruments, Trenton, NJ, USA). Cy3-dCTP, FluorX-dCTP, DEAC, Cy5-dCTP and DAPI fluorescence signals, detected with specific filters, were recorded separately as gray scale images. Pseudocoloring and merging of images were performed using Adobe Photoshop™ software.

### Library screening

STSs (Additional data file 1) used for CH250 high density filter library-screening were chosen from the University of California Santa Cruz database (UCSC; May 2004 release) [48]. Library screenings, using the human PCR products, were carried out according to a published protocol from Pieter De Jong [49]. The first segment of the CH250 macaque genomic library is 6.0× redundant [50]. The identification of additional BAC clones specific for rhesus macaque took advantage of specific genome browsers [40,48].

Fifteen overgo probes of 36-40 bp each were designed based on conserved sequences between the human and mouse genomes according to the HomoloGene database [51], using a previously described protocol [52]. The probes were hybridized to high-density filters of *F. catus *BAC libraries (RPCI-86; see Results) and the images were analyzed with ArrayVision Ver6.0 (Imaging Research Inc., Linton, UK). The sequence and location of overgo probes, along with clones they identified, are reported in Additional data file 2. Some overgo probes were also used to screen the rhesus macaque library (CHORI-250) to investigate the breakpoint regions (see Results).

Marker order reconstruction took advantage of the GRIMM software package, designed to outline the most parsimonious scenario of evolutionary marker order changes [53,54].

### Sequence and segmental duplication analyses

In order to show the sequence homology between the putative breakpoints, we analyzed segmental duplication pairwise alignments (size ≥10 kb and sequence identity ≥95%; build35 UCSC human genome Browser) defined by the whole genome assembly comparison method [55]. The ancestral origin of the duplications was determined as described [56]. The duplicons were color-coded based on the cytogenetic band location of their ancestral loci. The age of the duplications at each breakpoint was also estimated by calculating sequence identity between predicted ancestral and derived duplications.

## Abbreviations

BAC, bacterial artificial chromosome; DBR, distal breakpoint region; FISH, fluorescence *in situ *hybridization; PBR, proximal breakpoint region; STS, sequence tagged site.

## Authors' contributions

MFC carried out the molecular genetic studies, participated in the sequence alignment and drafted the manuscript. ZJ carried out the sequence alignment and performed the statistical analysis. PD participated in the sequence alignment. NA and MR participated in the design of the study. EEE participated in the sequence alignment, participated in the design of the study and performed critical reading and writing of the paper. MV conceived of the study, and participated in its design and coordination. All authors read and approved the final manuscript.

## Additional data files

The following additional data are available. Additional data file 1 is a table listing cat-specific BAC clones identified by library screening using overgo probes. Additional data file 2 is a table listing additional human BAC clones used to define the inversion breakpoints in macaque. Additional data file 3 is a table listing additional macaque BAC clones obtained by library screening or sequence analyses used to define the inversion breakpoints. Additional data file 4 is a figure showing a diagrammatic representation of the FISH signal of duplicated BAC clones. Additional data file 5 is a figure showing BAC clones from DUPA' and DUPB' on human and macaque metaphases as an example of FISH results. Additional data file 6 is a table listing pairwise sequence similarity between putative breakpoints.

## Supplementary Material

Additional data file 1 Cat-specific BAC clones identified by library screening using overgo probes. Click here for file

Additional data file 2 Additional human BAC clones used to define the inversion breakpoints in macaque. Click here for file

Additional data file 3 Additional macaque BAC clones obtained by library screening or sequence analyses used to define the inversion breakpoints. Click here for file

Additional data file 4 Each colored box indicates a different cluster of duplication detected by BAC clones: orange, BAC clone RP11-678G7 (Ed) from DUPA giving different hybridization patterns. See the text for details. Click here for file

Additional data file 5 BAC clones from **(a,b) **DUPA' and **(c,d) **DUPB' on human (a,c) and macaque (b,d) metaphases. White arrows indicate chromosome 17 and the homologous regions in macaque. Click here for file

Additional data file 6 Pairwise sequence similarity between putative breakpoints. Click here for file

## References

[B1] Müller S, Wienberg J (2001). "Bar-coding" primate chromosomes: molecular cytogenetic screening for the ancestral hominoid karyotype.. Hum Genet.

[B2] Murphy WJ, Eizirik E, Johnson WE, Zhang YP, Ryder OA, O'Brien SJ (2001). Molecular phylogenetics and the origins of placental mammals.. Nature.

[B3] Murphy WJ, Pevzner PA, O'Brien SJ (2004). Mammalian phylogenomics comes of age.. Trends Genet.

[B4] Murphy WJ, Stanyon R, O'Brien SJ (2001). Evolution of mammalian genome organization inferred from comparative gene mapping.. Genome Biol.

[B5] Richard F, Lombard M, Dutrillaux B (2003). Reconstruction of the ancestral karyotype of eutherian mammals.. Chromosome Res.

[B6] Wienberg J (2004). The evolution of eutherian chromosomes.. Curr Opin Genet Dev.

[B7] Yang F, Alkalaeva EZ, Perelman PL, Pardini AT, Harrison WR, O'Brien PC, Fu B, Graphodatsky AS, Ferguson-Smith MA, Robinson TJ (2003). Reciprocal chromosome painting among human, aardvark, and elephant (superorder Afrotheria) reveals the likely eutherian ancestral karyotype.. Proc Natl Acad Sci USA.

[B8] Carbone L, Ventura M, Tempesta S, Rocchi M, Archidiacono N (2002). Evolutionary history of chromosome 10 in primates.. Chromosoma.

[B9] Cardone MF, Ventura M, Tempesta S, Rocchi M, Archidiacono N (2002). Analysis of chromosome conservation in Lemur catta studied by chromosome paints and BAC/PAC probes.. Chromosoma.

[B10] Misceo D, Cardone MF, Carbone L, D'Addabbo P, de Jong PJ, Rocchi M, Archidiacono N (2005). Evolutionary history of chromosome 20.. Mol Biol Evol.

[B11] Kehrer-Sawatzki H, Schreiner B, Tänzer S, Platzer M, Müller S, Hameister H (2002). Molecular characterization of the pericentric inversion that causes differences between chimpanzee chromosome 19 and human chromosome 17.. Am J Hum Genet.

[B12] Szamalek JM, Goidts V, Searle JB, Cooper DN, Hameister H, Kehrer-Sawatzki H (2006). The chimpanzee-specific pericentric inversions that distinguish humans and chimpanzees have identical breakpoints in *Pan troglodytes *and *Pan paniscus*.. Genomics.

[B13] Zody MC, Garber M, Adams DJ, Sharpe T, Harrow J, Lupski JR, Nicholson C, Searle SM, Wilming L, Young SK, Abouelleil A, Allen NR, Bi W, Bloom T, Borowsky ML, Bugalter BE, Butler J, Chang JL, Chen CK, Cook A, Corum B, Cuomo CA, de Jong PJ, DeCaprio D, Dewar K, FitzGerald M, Gilbert J, Gibson R, Gnerre S, Goldstein S (2006). DNA sequence of human chromosome 17 and analysis of rearrangement in the human lineage.. Nature.

[B14] Bailey JA, Yavor AM, Viggiano L, Misceo D, Horvath JE, Archidiacono N, Schwartz S, Rocchi M, Eichler EE (2002). Human-specific duplication and mosaic transcripts: the recent paralogous structure of chromosome 22.. Am J Hum Genet.

[B15] Dörr S, Midro AT, Färber C, Giannakudis J, Hansmann I (2001). Construction of a detailed physical and transcript map of the candidate region for Russell-Silver syndrome on chromosome 17q23-q24.. Genomics.

[B16] Eichler EE, Johnson ME, Alkan C, Tuzun E, Sahinalp C, Misceo D, Archidiacono N, Rocchi M (2001). Divergent origins and concerted expansion of two segmental duplications on chromosome 16.. J Hered.

[B17] Samonte RV, Eichler EE (2002). Segmental duplications and the evolution of the primate genome.. Nat Rev Genet.

[B18] She X, Jiang Z, Clark RA, Liu G, Cheng Z, Tuzun E, Church DM, Sutton G, Halpern AL, Eichler EE (2004). Shotgun sequence assembly and recent segmental duplications within the human genome.. Nature.

[B19] She X, Liu G, Ventura M, Zhao S, Misceo D, Roberto R, Cardone MF, Rocchi M, Green ED, Archidiacano N, Eichler EE, NISC Comparative Sequencing Program (2006). A preliminary comparative analysis of primate segmental duplications shows elevated substitution rates and a great-ape expansion of intrachromosomal duplications.. Genome Res.

[B20] Chen DC, Saarela J, Clark RA, Miettinen T, Chi A, Eichler EE, Peltonen L, Palotie A (2004). Segmental duplications flank the multiple sclerosis locus on chromosome 17q.. Genome Res.

[B21] Lupski JR (1998). Genomic disorders: structural features of the genome can lead to DNA rearrangements and human disease traits.. Trends Genet.

[B22] Park SS, Stankiewicz P, Bi W, Shaw C, Lehoczky J, Dewar K, Birren B, Lupski JR (2002). Structure and evolution of the Smith-Magenis syndrome repeat gene clusters, SMS-REPs.. Genome Res.

[B23] Inoue K, Dewar K, Katsanis N, Reiter LT, Lander ES, Devon KL, Wyman DW, Lupski JR, Birren B (2001). The 1.4-Mb CMT1A duplication/HNPP deletion genomic region reveals unique genome architectural features and provides insights into the recent evolution of new genes.. Genome Res.

[B24] Lupski JR, de Oca-Luna RM, Slaugenhaupt S, Pentao L, Guzzetta V, Trask BJ, Saucedo-Cardenas O, Baker DF, Killian JM, Garcia CA, Chakravarti A, Patel PI (1991). DNA duplication associated with Charcot-Marie-Tooth disease type 1A.. Cell.

[B25] Potocki L, Chen KS, Koeuth T, Killian J, Iannaccone ST, Shapira SK, Kashork CD, Spikes AS, Shaffer LG, Lupski JR (1999). DNA rearrangements on both homologues of chromosome 17 in a mildly delayed individual with a family history of autosomal dominant carpal tunnel syndrome.. Am J Hum Genet.

[B26] Potocki L, Chen KS, Park SS, Osterholm DE, Withers MA, Kimonis V, Summers AM, Meschino WS, Anyane-Yeboa K, Kashork CD, Shaffer LG, Lupski JR (2000). Molecular mechanism for duplication 17p11.2- the homologous recombination reciprocal of the Smith-Magenis microdeletion.. Nat Genet.

[B27] Koolen DA, Vissers LE, Pfundt R, de Leeuw N, Knight SJ, Regan R, Kooy RF, Reyniers E, Romano C, Fichera M, Schinzel A, Baumer A, Anderlid BM, Schoumans J, Knoers NV, van Kessel AG, Sistermans EA, Veltman JA, Brunner HG, de Vries BB (2006). A new chromosome 17q21.31 microdeletion syndrome associated with a common inversion polymorphism.. Nat Genet.

[B28] Sharp AJ, Hansen S, Selzer RR, Cheng Z, Regan R, Hurst JA, Stewart H, Price SM, Blair E, Hennekam RC, Fitzpatrick CA, Segraves R, Richmond TA, Guiver C, Albertson DG, Pinkel D, Eis PS, Schwartz S, Knight SJ, Eichler EE (2006). Discovery of previously unidentified genomic disorders from the duplication architecture of the human genome.. Nat Genet.

[B29] Shaw-Smith C, Pittman AM, Willatt L, Martin H, Rickman L, Gribble S, Curley R, Cumming S, Dunn C, Kalaitzopoulos D, Porter K, Prigmore E, Krepischi-Santos AC, Varela MC, Koiffmann CP, Lees AJ, Rosenberg C, Firth HV, de Silva R, Carter NP (2006). Microdeletion encompassing MAPT at chromosome 17q21.3 is associated with developmental delay and learning disability.. Nat Genet.

[B30] Mefford HC, Clauin S, Sharp AJ, Moller RS, Ullmann R, Kapur R, Pinkel D, Cooper GM, Ventura M, Ropers HH, Tommerup N, Eichler EE, Bellanne-Chantelot C (2007). Recurrent reciprocal genomic rearrangements of 17q12 are associated with renal disease, diabetes, and epilepsy.. Am J Hum Genet.

[B31] Saarela J, Schoenberg Fejzo M, Chen D, Finnilä S, Parkkonen M, Kuokkanen S, Sobel E, Tienari PJ, Sumelahti ML, Wikström J, Elovaara I, Koivisto K, Pirttilä T, Reunanen M, Palotie A, Peltonen L (2002). Fine mapping of a multiple sclerosis locus to 2.5 Mb on chromosome 17q22-q24.. Hum Mol Genet.

[B32] Barbouti A, Stankiewicz P, Nusbaum C, Cuomo C, Cook A, Höglund M, Johansson B, Hagemeijer A, Park SS, Mitelman F, Lupski JR, Fioretos T (2004). The breakpoint region of the most common isochromosome, i(17q), in human neoplasia is characterized by a complex genomic architecture with large, palindromic, low-copy repeats.. Am J Hum Genet.

[B33] Armengol L, Pujana MA, Cheung J, Scherer SW, Estivill X (2003). Enrichment of segmental duplications in regions of breaks of synteny between the human and mouse genomes suggest their involvement in evolutionary rearrangements.. Hum Mol Genet.

[B34] Bailey JA, Baertsch R, Kent WJ, Haussler D, Eichler EE (2004). Hotspots of mammalian chromosomal evolution.. Genome Biol.

[B35] Bailey JA, Church DM, Ventura M, Rocchi M, Eichler EE (2004). Analysis of segmental duplications and genome assembly in the mouse.. Genome Res.

[B36] Murphy WJ, Larkin DM, Everts-van der Wind A, Bourque G, Tesler G, Auvil L, Beever JE, Chowdhary BP, Galibert F, Gatzke L, Hitte C, Meyers SN, Milan D, Ostrander EA, Pape G, Parker HG, Raudsepp T, Rogatcheva MB, Schook LB, Skow LC, Welge M, Womack JE, O'brien SJ, Pevzner PA, Lewin HA (2005). Dynamics of mammalian chromosome evolution inferred from multispecies comparative maps.. Science.

[B37] Stankiewicz P, Park SS, Inoue K, Lupski JR (2001). The evolutionary chromosome translocation 4;19 in *Gorilla gorilla *is associated with microduplication of the chromosome fragment syntenic to sequences surrounding the human proximal CMT1A-REP.. Genome Res.

[B38] Ventura M, Archidiacono N, Rocchi M (2001). Centromere emergence in evolution.. Genome Res.

[B39] Ventura M, Mudge JM, Palumbo V, Burn S, Blennow E, Pierluigi M, Giorda R, Zuffardi O, Archidiacono N, Jackson MS, Rocchi M (2003). Neocentromeres in 15q24-26 map to duplicons which flanked an ancestral centromere in 15q25.. Genome Res.

[B40] Rhesus Macaque (*Macaca mulatta*) BAC Mapping Via PGI. http://brl.bcm.tmc.edu/pgi/rhesus/.

[B41] Bailey JA, Eichler EE (2006). Primate segmental duplications: crucibles of evolution, diversity and disease.. Nat Rev Genet.

[B42] Gibbs RA, Rogers J, Katze MG, Bumgarner R, Weinstock GM, Mardis ER, Remington KA, Strausberg RL, Venter JC, Wilson RK, Batzer MA, Bustamante CD, Eichler EE, Hahn MW, Hardison RC, Makova KD, Miller W, Milosavljevic A, Palermo RE, Siepel A, Sikela JM, Attaway T, Bell S, Bernard KE, Buhay CJ, Chandrabose MN, Dao M, Davis C, Delehaunty KD, Rhesus Macaque Genome Sequencing and Analysis Consortium (2007). Evolutionary and biomedical insights from the rhesus macaque genome.. Science.

[B43] Wang NJ, Parokonny AS, Thatcher KN, Driscoll J, Malone BM, Dorrani N, Sigman M, Lasalle JM, Schanen NC (2008). Multiple forms of atypical rearrangements generating supernumerary derivative chromosome 15.. BMC Genet.

[B44] Conrad B, Antonarakis SE (2007). Gene duplication: a drive for phenotypic diversity and cause of human disease.. Annu Rev Genomics Hum Genet.

[B45] Ji Y, Eichler EE, Schwartz S, Nicholls RD (2000). Structure of chromosomal duplicons and their role in mediating human genomic disorders.. Genome Res.

[B46] Cáceres M, Sullivan RT, Thomas JW, National Institutes of Health Intramural Sequencing Center Comparative Sequencing Program (2007). A recurrent inversion on the eutherian X chromosome.. Proc Natl Acad Sci USA.

[B47] Lichter P, Tang CJ, Call K, Hermanson G, Evans GA, Housman D, Ward DC (1990). High-resolution mapping of human chromosomes 11 by *in situ *hybridization with cosmid clones.. Science.

[B48] UCSC Genome Browser. http://genome.ucsc.edu.

[B49] CHORI Resources: Hybridization of High Density Filters. http://bacpac.chori.org/highdensity.htm.

[B50] CHORI Resources: CH250 Rhesus Macaque BAC Library. http://bacpac.chori.org/rhesus250.htm.

[B51] HomoloGene Home. http://www.ncbi.nlm.nih.gov/sites/entrez/query.fcgi?db=homologene.

[B52] McPherson JD, Marra M, Hillier L, Waterston RH, Chinwalla A, Wallis J, Sekhon M, Wylie K, Mardis ER, Wilson RK, Fulton R, Kucaba TA, Wagner-McPherson C, Barbazuk WB, Gregory SG, Humphray SJ, French L, Evans RS, Bethel G, Whittaker A, Holden JL, McCann OT, Dunham A, Soderlund C, Scott CE, Bentley DR, Schuler G, Chen HC, Jang W, Green ED (2001). A physical map of the human genome.. Nature.

[B53] Bourque G, Pevzner PA (2002). Genome-scale evolution: reconstructing gene orders in the ancestral species.. Genome Res.

[B54] Genome Rearrangements in Man and Mouse. http://grimm.bioprojects.org/GRIMM/.

[B55] Bailey JA, Yavor AM, Massa HF, Trask BJ, Eichler EE (2001). Segmental duplications: organization and impact within the current human genome project assembly.. Genome Res.

[B56] Jiang Z, Tang H, Ventura M, Cardone MF, Marques-Bonet T, She X, Pevzner PA, Eichler EE (2007). Ancestral reconstruction of segmental duplications reveals punctuated cores of human genome evolution.. Nat Genet.

